# Community residents’ preferences for chronic disease management in Primary Care Facilities in China: a stated preference survey

**DOI:** 10.1186/s13690-021-00728-8

**Published:** 2021-11-26

**Authors:** Xianglin Li, Mingzhu Jiang, Yingying Peng, Xiao Shen, Erping Jia, Juyang Xiong

**Affiliations:** The School of Medicine and Health Management, Tongji Medical College, Huazhong University of Science and Technology, 13 Hangkong Road, Wuhan, 430030 Hubei China

**Keywords:** Chronic diseases, Primary care, Healthcare, China, Discrete choice experiment

## Abstract

**Background:**

Although Chinese government has dedicated the past decades to treating chronic diseases by primary healthcare system, many more residents are apt to choose higher-tier facilities to treat minor chronic diseases. Understanding residents’ preferences for chronic disease management in primary care facilities can bridge the gap between residents’ choices and policy implementation. This study aims to elicit residents’ preferences for chronic disease management in primary care facilities in the hypothetical minor chronic disease scenario.

**Methods:**

Six hundred eighty residents were administered a discrete choice experiment that elicited preferences for chronic disease management in primary care facilities. Services attributes were service mode, treatment measure, out-of-pocket expenditure (OOP), traveling time to healthcare facility and title of physician. Mixed logit models were used to estimate stated preferences and willingness to pay for attributes. WTP confidence intervals were estimated by the delta method.

**Results:**

A total of 94.44% of the completed questionnaires were valid (680 of 720 respondents). The participants preferred chronic disease management service with modern medicine, traveling time ≤ 30mins, and less OOP expenditure. Compared with Traditional Chinese Medicine (TCM), residents prefer modern medicine, willing to pay 155.53 CNY ($21.97) to change from TCM to modern medicine. Compensation about 86.02 CNY ($12.15) was needed to enable residents to change the choice of the nearer primary care facility to a further one. Integrated medicine in community clinics by experts was residents’ most preferred scenario while TCM in the tertiary hospital was their least preferred one.

**Conclusion:**

In order to increase the utilization of primary healthcare services in chronic diseases management, policy makers need to concern more about the services of medical treatment type, price and convenience. Therefore, we advise policy makers to provide nearer primary healthcare services for residents especially for residents in surrounding areas. Furthermore, balancing the resource allocation between Traditional Chinese Medicine and modern medicine is worthy of consideration.

**Supplementary Information:**

The online version contains supplementary material available at 10.1186/s13690-021-00728-8.

## Background

Chronic disease is one of the major health concerns in the world. World Health Organization reported that about 41 million people died of various chronic diseases in 2016, accounting for 71% of the total deaths [1]. Also, chronic diseases caused heavy financial burdens for payers worldwide [2]. In China, a national report stated that chronic diseases were attributable to 88.5% of all deaths in 2019 [3]. It was estimated that the interventions associated with chronic diseases were attributable to about 70% of the total health expenditures [4].

There is a consensus that primary care facilities are crucial for managing chronic diseases in a sustainable way, which could reduce the financial burdens and improve the health outcomes of chronic diseases [5]. Following the lessons from western countries, China has established a de facto multi-tiered healthcare system (i.e., tertiary hospitals, secondary hospitals, and primary care facilities) with an emphasis on the role of primary care facilities (e.g., community health centers, clinics) in chronic disease management [6]. Although the number of primary care facilities has increased from 676,483 to 943,639 and the proportion of healthcare practitioners with a bachelor’s degree working in community health centers has increased from 21.9% to 48.4% during last few years [7, 8], the utilization of primary care facilities even decreased from 57.76% in 2014 to 53.04% in 2018 [8]. It reflects the unexpected fact that residents show less preference for chronic disease management in primary care facilities [9].

Previous studies have conducted surveys to investigate the factors that influence the community residents’ choice of primary care facilities for chronic disease management. Distance to practice [10], price, medical insurance [11], trust in care providers [12], and parking [13] were residents concern. However, there is limited quantitative evidence surrounding the residents’ preferences. It is unclear how the residents assess the factors that may influence their choices and how they make decisions and trade-offs.

The objective of this study is to elicit community residents’ preferences for chronic disease management in primary care facilities in Wuhan, China, and estimate their willingness-to-pay (WTP) in the context of different policy scenarios.

## Methods

We conducted a discrete choice experiment (DCE) among a sample of community residents in Wuhan, China, to elicit their preferences for primary care facilities in chronic disease management. The DCE is a broadly accepted experimental method based on random utility theory that elicits respondents’ stated preferences for a good or service [14]. A DCE choice task presents respondents with two or more hypothetical goods/services (called profiles) described by a series of characteristics (called attributes) and a question requiring respondents to select the one that yields the largest utility among the several profiles from their perspectives. The responses are used to establish limited dependent variable regression models that yield estimates on the preference values that respondents assign on the attribute levels.

### DCE design

We started the DCE by identifying the attributes associated with the selection of healthcare facilities. Through a scoping review of the literature (see Appendix 1 in Supplementary Materials and Methods), we identified factors that may affect the residents’ choices for primary care facilities, including service mode, out-of-pocket expenditure (OOP), the location of first appointment, traveling time to the healthcare facility, type of the physician, the opening hours, type of the insurance, information accessibility, patient involvement in decision-making, and the follow-up arrangements [15]. As modern medicine and Traditional Chinese Medicine (TCM) are practiced alongside each other at every level of the health care system in China [16]. We also considered the availability of Traditional Chinese Medicine (TCM) and integrated medicine (i.e., TCM and modern medicine combined service) in addition to modern medicine service in the attribute of medical treatment type.

We conducted two rounds of focus group consultation in Wuhan, China (see Table S1 in Supplementary Materials and Methods). The first group was composed of 2 scientists in health management and policy, 2 directors of primary care facilities, and 3 primary care practitioners, who were invited in November 2017. The second group consisted of 10 residents from Qiaokou municipality, who were invited in March 2018. By collecting their opinions on the factors that we previously identified from the literature, 5 attributes and associated levels were finally identified (Table 1).

**Table 1:** Discrete choice experiment attributes and levels

**Scenario**	**Level**	**Explanation**
Hypothetical perceived disease severity	Minor	The perceived minor chronic diseases cause occasional discomfort and does not seriously affect daily life.
**Attributes**	**Explanation**	**Levels**
Service mode	Different modes of services chosen by residents when they are registered.	Specialized service General service
Medical treatment type	Different forms of medical treatment.	Traditional Chinese Medicine (TCM)
		Modern medicine
		Integrated medicine
Out-of-pocket expenditure (OOP) (CNY, Chinese Yuan)*	Individuals actually pay the average expense of each visit for primary care services.	100
		200
		300
Traveling time to the healthcare facility	The time taken to go to the facility from home (one-way travel).	≤30mins
		>30mins
Type of the physician	The seniority of the individual in the facility.	Junior healthcare practitioner
		Senior healthcare practitioner

*OOP was set according to the data of China Health Statistical Yearbook 2018 [8]

### Sampling

We used a multi-stage sampling method, which included three stages. The first stage was stratified random sampling for municipalities. Since Wuhan is a metropolis with 7 central municipalities and 6 surrounding municipalities. We randomly selected a central municipality (Qiaokou) and a surrounding municipality (Jiangxia) for participant recruitment in order to cover areas with differences in healthcare resources. (see Table S2 in Supplementary Materials and Methods).

The second stage was random sampling for community health centers. There are 36 and 27 community health centers in Qiaokou municipality and Jiangxia municipality respectively. We numbered the community health centers in the two municipalities respectively, and randomly selected 6 of each municipality for the next stage of sampling.

The third stage is quota sampling for residents. As sample size estimation methods in healthcare DCE studies are currently developing [17], we chose the Johnson and Orme rule [18, 19] of thumb equation to determine the minimum acceptable sample size yielding a recommended minimum sample size of 150. Much larger sample was targeted to allow for heterogeneity between respondents [20], we aimed to recruited a total of 720 respondents from 12 facilities in Wuhan, each facility recruited 60 residents. The inclusion criteria included that: 1) the respondent lived in communities nearby for more than half a year, 2) the age was no less than 18 years old, 3) the respondent was capable to read and understand Chinese, and 4) the respondent provided informed consent to participate in the survey. According to the local population, males and the population aged 18–45 accounted for 51 and 52.49% respectively [21]. Therefore, we pre-defined the sample quota on gender (31 men and 29 women) and age (29 of 18-35 years and 31 of > 45 years). The administrative staffs in each primary care facility assisted in making invitation calls to residents and made appointments with those who agreed to participate. The recruitment ended until we achieved the goal of sample size.

### Questionnaire design

**Fig. 1:**
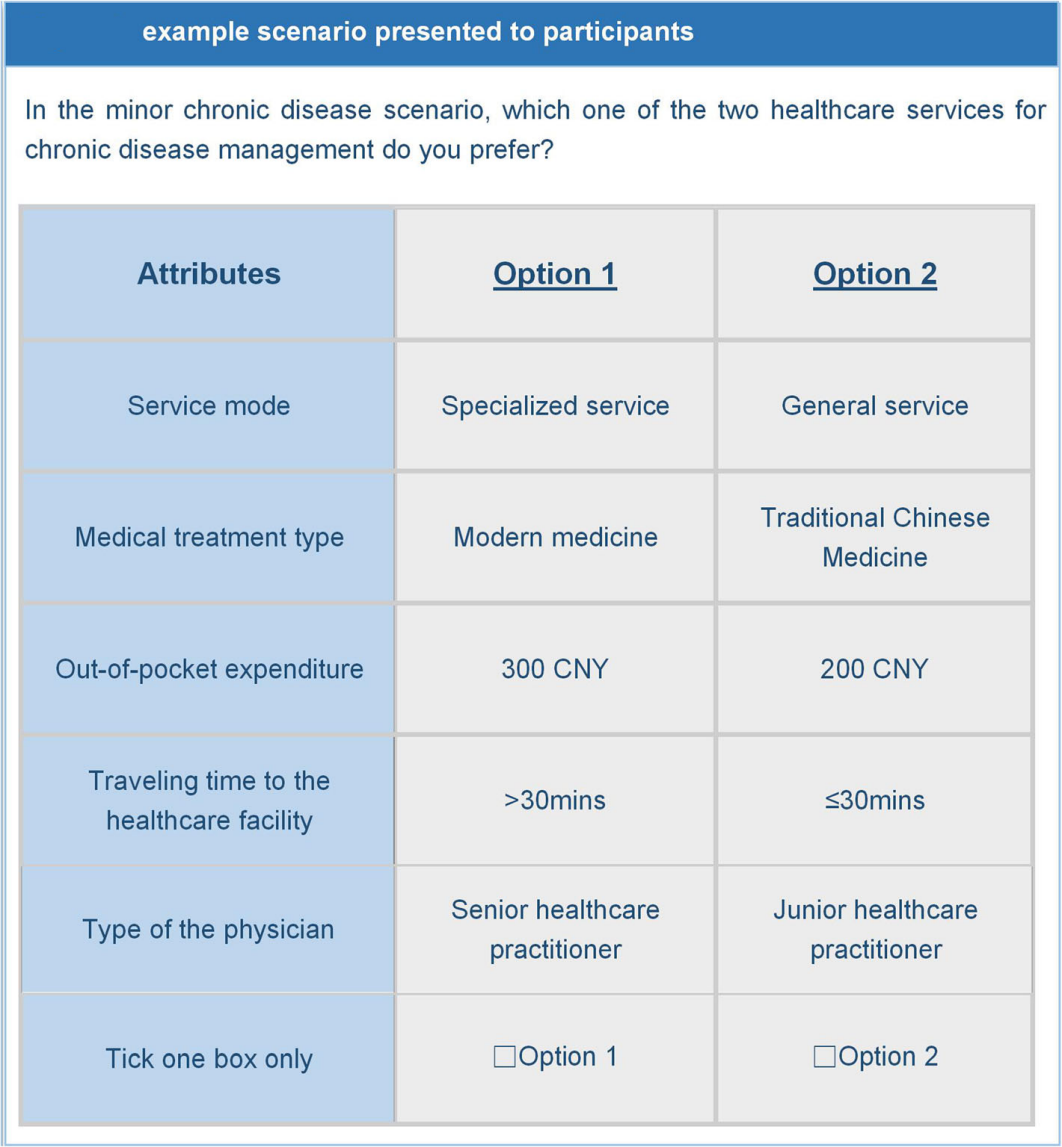
Example Scenario presented to participants

We applied an orthogonal experimental design in SPSS (IBM Corp. Version 22.0. Armonk, NY) and generated 16 choice tasks. To further reduce the cognitive burden of respondents, we divided the 16 choice tasks into two blocks, each with 8 choice tasks, and produced two versions of questionnaires accordingly [22]. We randomly assigned the two versions among respondents to ensure that the numbers of participants using two versions were balanced. Each choice task was comprised of two hypothetical profiles, each of which was composed by levels respectively from the five attributes we identified. In each choice task, respondents were asked to select the preferred profile that hypothetically described a healthcare facility (Fig. 1). We didn’t leave respondents an opt-out option. Given that respondents with uncertain choices may choose opt-out option to avoid making difficult decisions, a forced choice can guarantee data with better quality [23]. We included a rationality test in the questionnaire by setting a dominant choice task (i.e., the choice task included a profile composed by logically preferable levels on all attributes). If respondents didn’t choose the dominant profile, we decided that the respondent failed the test and excluded his/her response. In addition, we included some demographic questions such as gender, age, and marital status in the questionnaire.

### Pilot test

A paper-based pilot test was administered to 50 residents in March 2018 in Qiaokou municipality, Wuhan (see Table S3 and Table S4 in Supplementary Materials and Methods). Respondents completed the questionnaires without time limitation. During the pilot test, we also communicated with respondents about their opinions on the attributes and possible difficulties and confusions in understanding the questionnaire. According to their feedback, we improved the format and language to make the questionnaire more understandable.

### Survey administration

We conducted a face-to-face, one-on-one interview with each respondent. To facilitate the interview, trained interviewers would assist respondents by introducing the interview procedure, obtaining written consent forms, explaining the definitions of attributes and associated levels, and describing the example choice task. The respondents were expected to complete the questionnaire by themselves, and they were allowed to ask questions during the interview. Primary care facilities provided separate rooms for the interviews. After the interview, we provided a gift worthy of 50 CNY ($7.13) to respondents. The survey data was collected from May 2018 to August 2018. The study was approved by the Ethics Committee of Tongji Medical College, Huazhong University of Science and Technology (IORG No: IORG0003571) on December 25, 2016.

### Statistical analysis

Data were analyzed in STATA 15.1 (StataCorp LP) using a mixed logit model. We specified the coefficients of OOP-associated levels as fixed to avoid implausibly large values and heavily skewed distributions of willingness-to-pays (WTPs). The other attributes were categorical and effects-coded. We specified the coefficients of the attribute levels excluding OOP as random parameters following normal distributions. WTPs were calculated by dividing the coefficients of non-cost attribute levels by the coefficient of OOP. WTP confidence intervals were estimated using the delta method [24].

## Results

### Participant demographics

We invited 807 residents, among whom 720 agreed to participate (participation rate 89.2%). A total of 680 participants completed the questionnaires (response rate 84.3%), among which 349 were residents in the central municipality and 331 were in the surrounding municipality.

The majority of our respondents were female. The mean age of respondents was 48 years. Most of the respondents were married and had medical insurance. In terms of educational level, respondents with a high school level were about twice as those with an elementary school level or below. Nearly half of the respondents were employed. Over 70% of the respondents had no chronic disease (Table 2).

**Table 2:** Demographics from residents

Demographics	Sample	Wuhan census^**a**^
Gender (Male, %)	206 (30.29)	51.0
Age (18–45, %)	311 (45.74)	47.51
Marital status (Married, %)	576 (84.71)	─
Educational level (Elementary school and below, %)		
High school	297 (43.68)	26.12
Undergraduate and above	202 (29.71)	30.28
Occupation (Unemployed, %)		
Employed	310 (45.59)	66.08
Retired	215 (31.62)	
Area (Central municipality, %)	349 (51.32)	29.10
Insurance coverage (Yes, %)	654 (96.18)	95.75
Monthly income (CNY) [Median (IQR^b^)]	3000 (2000, 5000)	3102.92
Quantity of chronic diseases (0, %)		
1	148 (21.76)	24.52
≥ 2	48 (7.10)	

^a^The data of gender, age, occupation, area and monthly income was from Wuhan Statistical Yearbook 2018 [21]. The data of educational level was from Tabulation on the Population census of Hubei [25]. The data of insurance coverage and prevalence of chronic diseases was from China Health Statistical Yearbook 2018 [8]. No official data found for marital status

### Mixed logit model estimates

**Fig. 2:**
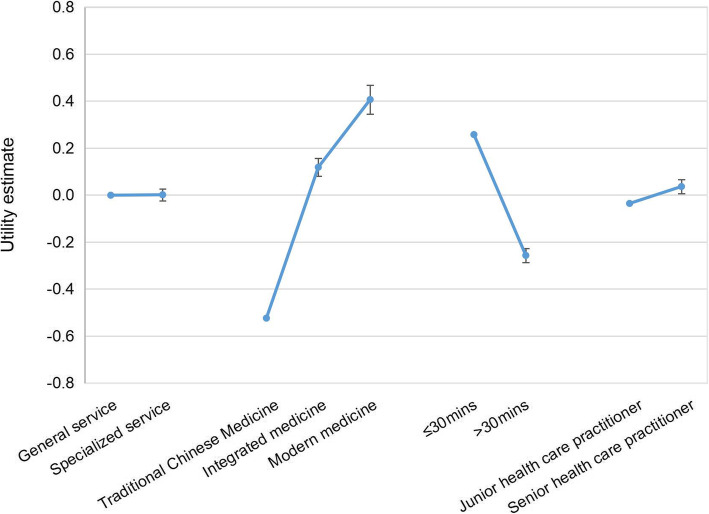
Residents’ preference weight for chronic disease management in primary care facilities

The estimates of the mixed logit model are summarized in Table 3 and illustrated in Fig. 2. The coefficients of levels of three attributes were statistically significant. We found respondents preferred healthcare services provided by senior practitioners ((Mean = 0.001 (SE = 0.025)) in care facilities with a traveling time less than 30 min ((Mean = 257 (SE = 0.033)). They also preferred modern medicine service ((Mean = 0.406 (SE = 0.062)) and integrated medicine ((Mean = 0.118 (SE = 0.038)) to TCM ((Mean = − 0.524 (SE = 0.071)). They were less likely to accept care services provided by junior practitioners in primary care facilities with a traveling time more than 30 min.

**Table 3:** Results from mixed logit models of DCE data

Attributes	Levels	Mean^**a**^	SE	SD	SE	Preference value > 0, %
Service mode	General service (ref)	−0.001	0.026	─	─	─
	Specialized service	0.001	0.025	0.199 *	0.057	50.2
Medical treatment type	TCM service (ref)	−0.524*	0.071	─	─	─
	Modern medicine service	0.406*	0.062	1.312*	0.072	62.14
	Integrated medicine	0.118*	0.038	0.032	0.181	99.98
Traveling time to the healthcare facility	≤30mins (ref)	0.257*	0.033	─	─	─
	>30mins	−0.257*	0.03	0.459 *	0.04	28.75
Type of the physician	Junior health care practitioner (ref)	−0.036	0.035	─	─	─
	Senior health care practitioner	0.036	0.03	0.528 *	0.042	52.79
OOP (CNY)		−0.006*	0.001	─	─	─

Abbreviation: *Mean* coefficient means, *SE* standard error, *SD* standard deviation, *WTP* willingness to pay, *ref*. reference level

^a^Coefficients of the reference levels are calculated as the negative sum of the coefficients of the other levels of the attribute

^*^p < 0.05

Number of respondents =680, number of observations =10,880. Log-likelihood ratio of the mixed logit model = − 3050.103

Preference value> 0 (%): given the mean and SD of the coefficient, the proportion of respondents assigned a positive preference value on the service associated with the specified attribute level

### Relative importance of attributes

**Fig. 3:**
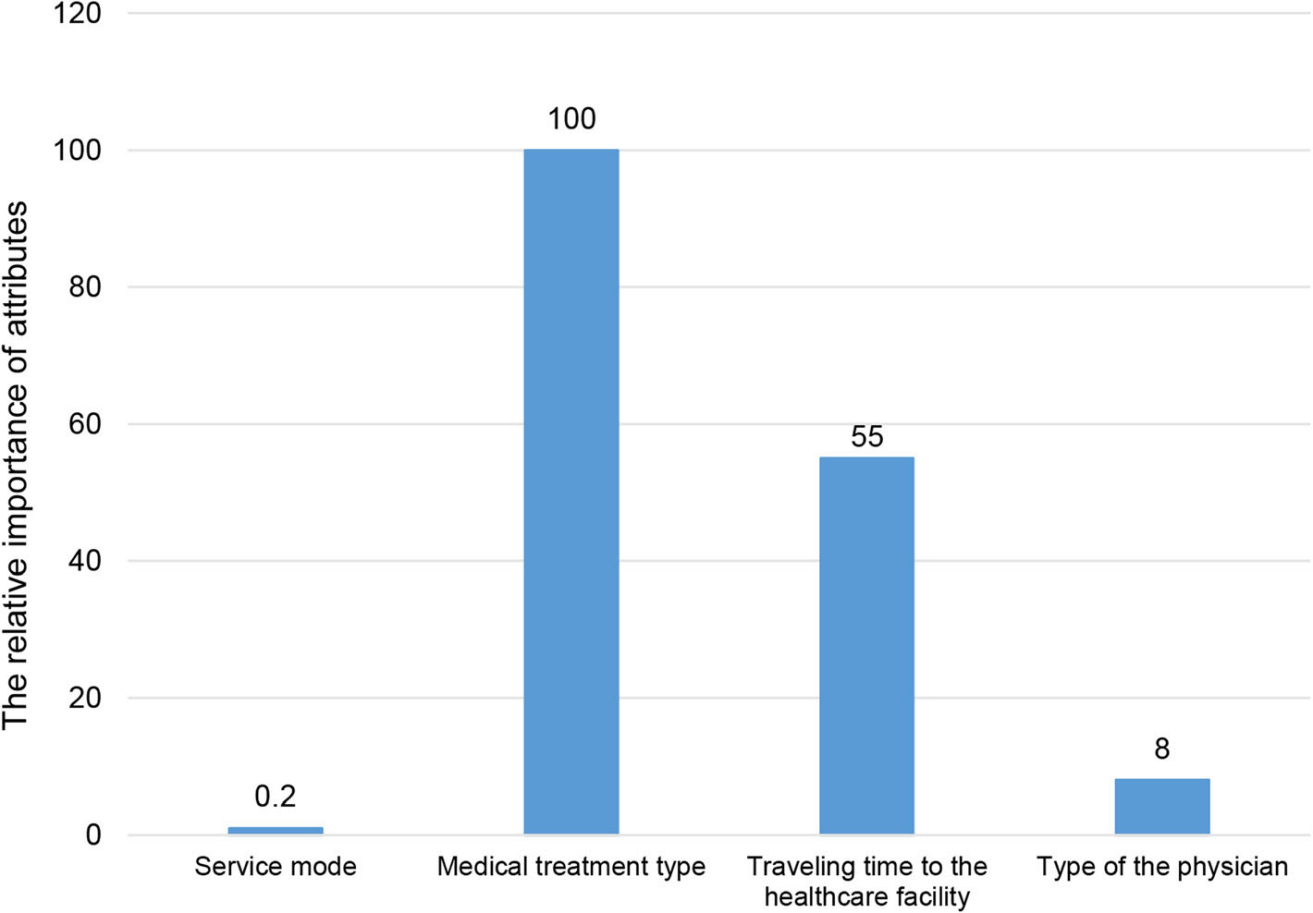
Relative importance of attributes in the DCE

The relative importance of attributes is illustrated in Fig. 3. The most important attribute was the medical treatment type, followed by traveling time to the healthcare facility and type of the physician were in the middle. The least important one was the service mode.

### Preference heterogeneity for attribute levels

We observed preference heterogeneity in most attributes (Table 3). For example, about 50% respondents placed negative reference value on specialized service. Although the majority of respondents were inclined to choose modern medicine, there were still 37.86% respondents placed negative values on this attribute level. It is worth noting that most of the respondents (99.98%) placed positive value on integrated medicine.

### Results from subgroup analysis

Subgroup analysis was conducted based on the municipality disparities, the coefficients displayed similar results in each term of size and significance and were with results of main effect model (see Table S4 in Supplementary Materials and Methods).

### Willingness-to-pay for healthcare services

The WTP related to the change from TCM service to modern medicine service was the largest, residents were willing to pay 155.53 CNY ($21.97). Residents were willing to pay 107.41 CNY ($15.17) to change from TCM service to integrated medicine service. Compensation about 86.02 CNY ($12.15) was needed to enable residents to change the choice of the nearer primary healthcare facility to a further one.

According to the results from subgroup analysis, residents in central municipality were willing to pay 155.33 CNY ($22.12) to change from TCM service to modern medicine service while residents in surrounding municipality were willing to pay 157.5 CNY ($22.44). Compensation about 54.38 CNY ($7.75) and 116.58 CNY ($12.62) was needed to enable residents in central municipality and residents in surrounding municipality to give up the choice of the nearer primary care facility (See Table S4 in Supplementary Materials and Methods).

We estimate residents’ WTP for chronic disease management in the context of different scenarios (See Table S5 in Supplementary Materials and Methods). In the base scenario (tertiary hospital), residents were willing to pay 32.69 CNY ($4.62). In the most preferred scenario (Integrated medicine in community clinic by experts), residents were willing to pay 67.30 CNY ($9.51). While compensation about 122.86 CNY ($17.36) was needed to enable residents to choose the least preferred scenario (TCM in tertiary hospital).

## Discussion

This study identifies community residents’ preferences for chronic disease management in primary care facilities in Wuhan, China, and their willingness to pay for chronic disease management services in different policy-relevant scenarios. The participants exhibited strong preferences for chronic disease management service with modern medicine, traveling time ≤ 30mins, and less OOP expenditure. Compared with TCM, they prefer modern medicine and integrated medicine, willing to pay 155.53 CNY ($21.97) and 107.41 CNY ($15.17) to change from TCM treatment to modern medicine and integrated medicine respectively. Residents preferred service with traveling time ≤ 30mins to time > 30mins, willing to pay 86.02 CNY ($12.03) change from a long time to a short time. Integrated medicine in community clinics by experts was residents’ most preferred scenario while TCM in the tertiary hospital was their least preferred one.

Among all type of medical treatment, the modern medicine was preferable relative to other types. As compared with TCM, modern medicine is generally considered to alleviate the condition quickly, which can partly explain residents’ preference for modern medicine especially in the minor chronic disease scenario [26]. Moreover, Residents have a consistent preference for integrated medicine. This may be linked to their strong beliefs in the complementary nature of TCM and modern medicine [27]. In addition, more clinical results indicate the synergistic use of both treatment measures to manage chronic diseases would yield a better health outcome than using either one alone [28, 29]. Therefore, residents with chronic diseases are more likely to choose integrated medicine, expecting to maximize the curative effects. The treatment of “the family doctor team of integrated medicine service” is worth considering to provide in primary care facilities, which not only attract residents to retain but also provides with better personalized and targeted primary healthcare services [30].

OOP and distance to practice also have large impact on the preferences. The results were in line with Liu.Y [31] and Sun.X [6]. This can be associated with residents’ favor of cheap and convenient health care services [31]. In China, one of health policies goals is to provide residents with convenient and affordable primary health care services [32], which is also in line with residents’ preferences in our study. It can be expected that with achievement on this goal, residents are very likely to choose primary care facilities in minor chronic diseases scenario.

Although residents prefer services with less OOP expenditure and traveling time, the majority of them are willing to pay 86.02 CNY ($12.03) to change from a long time to a shorter time. The concern regarding the high importance attached to traveling time can be linked to the complexity of chronic diseases. As delays may lead to a lack of appropriate healthcare, which can exacerbate health outcomes [33]. The rational allocation of health resources can counteract the residents’ behavior of seeking healthcare services [34], suggesting that decision makers should promote the rational layout of health care facilities about space and quantity [35], ensuring the homogeneous and geographically accessible primary healthcare services for residents. For groups with less sensitivity to traveling time, improving financial accessibility is critical. It implies that reducing the OOP expenditure of healthcare services will become an advantageous means for residents to receive primary healthcare services.

Residents especially those between central and surrounding municipalities have different willingness to pay for traveling time. Compared with the central municipality, residents in the surrounding municipality are willing to pay 24 CNY ($3.42) more to choose services with short traveling time from home. This can be explained by the primary care facilities having not yet been fully configured in the surrounding municipality. Since one public healthcare facilities provides health services to residents within an area of 0.11 km^2^ in Qiaokou, but within an area of 3.58 km^2^ in Jiangxia [21, 36]. Therefore, more primary care facilities should be strategically set up in the surrounding municipality to improve the structural accessibility of healthcare services. Moreover, as telemedicine can overcome geographical barriers and increase access to healthcare services [21], it can be fully utilized to suit residents’ practical needs for convenient primary healthcare services.

This study has several limitations. First, WTP we calculated was an estimate of the relative utility of services but not the true WTP. Second, due to limited sample sizes, we only investigated whether preferences for primary care facilities varied between central and surrounding municipalities. We did not investigate whether preferences varied across other subgroups such as educational level. Third, though we had 680 respondents who were community residents, it may not be able to broadly represent the population in Wuhan, which is a major target city for population migration in China. By recognizing that we were not able to capture the preferences of those migrants, it should be cautious to generate policy implications completely based on this study.

## Conclusion

The findings of this study have implications for the allocation of primary healthcare resources. Based on the respondents’ preferences, a conclusion can be drawn that residents generally considered factors concerned with the medical treatment type, price, and convenience. To guide residents to receive chronic disease management services in primary care facilities, the above basic needs were suggested to be fulfilled. Providing closer primary healthcare services for residents especially for those in surrounding areas and balancing the resource allocation between TCM and modern medicine is worthy of consideration by policy makers.

## Supplementary Information

**Additional file 1.**

## Data Availability

The datasets used and/or analyzed during the current study are available from the corresponding author on reasonable request.

## Abbreviations

TCM: Traditional Chinese Medicine
DCE: Discrete choice experiment
OOP: Out-of-pocket expenditure
SE: standard error
SD: standard deviation
WTP: willingness to pay
