# Supplementary material for: Community residents’ preferences for chronic disease management in Primary Care Facilities in China: a stated preference survey
Source: Arch Public Health. 2021 Nov 26;79:211. doi: 10.1186/s13690-021-00728-8 (PMC8620165; doi:10.1186/s13690-021-00728-8)
Supplement: Supplementary file 1 — Additional file 1. [file 13690_2021_728_MOESM1_ESM.docx]

**Appendix 1**

**Literature review**

Literature review was conducted to choose the most factors affecting residents using primary health care services. We searched Web of Science, Pubmed and Elsevier for English language articles, and three large Chinese databases (CNKI, VIP and Wanfang) for articles in Chinese. For obtaining a recent overview of the literature, we searched for studies published in the last 10 years (2007-2017). Electronic searches for relevant databases were conducted on November 2017. The search included: (1) terms related to primary healthcare services; (2) terms related to influential factors for residents’ choice of primary healthcare services; (3) terms related to chronic diseases. Search terms were: (“public preference” OR “public priorities” OR “influential factors”) AND (“primary healthcare” OR “medical treatment” OR “health-seeking behaviors”) AND (“Chronic diseases” OR “non-communicable diseases OR “NCDs”). We focused on systematic literature reviews and articles with higher impact factors to finally select factors that affect residents’ choices for primary health care services.

**Appendix 2**

**Table S1. Focus groups**

| **Group** | **No. Participants** | **Tasks and Content** | **Results** |
| --- | --- | --- | --- |
| 1 | 7  (Mean age of 42.9 years, range 29-57, SD=8.8 years.57% were male.) | An expert consultation focused on which attributes were the most influential when residents choose primary healthcare services for chronic disease management. Experts were invited to rank the importance of ten attributes from the literature review. | 6 attributes were remained into consideration, including: service mode, out-of-pocket expenditure (OOP), traveling time to the healthcare facility, type of the physician, patient involvement in decision-making, and medical treatment type.  Levels of each attribute were set as followed: The type of the physician included junior healthcare practitioner and senior healthcare practitioner. The out-of-pocket expenditure included 100 CNY ($14.30), 200 CNY ($28.60), 300CNY ($42.91). The medical treatment type included Traditional Chinese Medicine, modern medicine, and integrated medicine. The traveling time to healthcare faility included travel time ≤30 mins and >30 mins. The service mode included general service and specialized service. The patient involvement in decision-making included patient not involved and patient involved. |
| 2 | 10  (Mean age of 46.1 years, range 29-66, SD=12.2 years. 50% were male. ) | A focus group discussion guided by semi structured focus group guide.  Participants evaluate attributes and levels filtered by experts to determine which attributes and levels are appropriate to be remained. | Participants suggested attribute of shared decision making should be excluded. They considered this attribute level could be changed with factors such as residents’ moods and care provider’s attitude, which made it difficult to estimate their actual preferences. |

**Appendix 3**

**Table S2. Healthcare resources in sample areas**

| **area** | **Resident population^a^** | **Number of hospital**  **per 1000 population^b^** | **Number of primary care facilities per 1000 population** | **Number of medical technical personnel per 1000 population** | **Number of beds per 1000 population** |
| --- | --- | --- | --- | --- | --- |
| **Central municipality** | 6758800 | 0.041 | 0.411 | 13.309 | 10.757 |
| **Qiaokou** | 868900 | 0.06 | 0.36 | 22.017 | 17.764 |
| **Surrounding municipality** | 4453200 | 0.025 | 0.662 | 4.989 | 5.018 |
| **Jiangxia** | 987000 | 0.026 | 0.539 | 3.331 | 2.068 |
| **Wuhan** | 4453200 | 0.034 | 0.511 | 10.004 | 8.594 |

a The data of resident population was from Wuhan Statistical Yearbook 2020 [36].

b The data of number of hospital, primary care facilities, medical technical personnel, beds was from Wuhan Health Statistical Yearbook 2020 [38]. .

**Appendix 4**

**Table S3. Demographics from residents in the pilot test**

| **Demographics** | **Sample** |
| --- | --- |
| Gender (Male, %) | 23 (46) |
|  |  |
| Age (18-45, %) | 29 (58) |
|  |  |
| Marital status (Married, %) | 36 (72) |
|  |  |
| Educational level (Elementary school and below, %) |  |
| High school | 24 (48) |
| Undergraduate and above | 17 (34) |
|  |  |
| Occupation (Unemployed, %) |  |
| Employed | 35 (70) |
| Retired | 7 (14) |
|  |  |
| Area (Uptown, %) | 25 (50) |
|  |  |
| Insurance coverage (Yes, %) | 38 (96) |
|  |  |
| Monthly income (CNY) [Median (IQR^b^)] | 4000 (2550, 6000) |
|  |  |
| Quantity of chronic diseases (0, %) |  |
| 1 | 8 (16) |
| ≥2 | 6 (12) |

**Table S4. Residents’ suggestions in the pilot test**

| **No.**  **Participants** | **Tasks and Content** | **Suggestions** |
| --- | --- | --- |
| 50 | Participants were required to complete the questionnaire and give us advice on the questionnaire. | The questionnaire was thought to be appropriate in length.  The wording need to be more brief and comprehensible.  The explanation of each attribute and example question need to be added. |

**Appendix 5**

**Table S5. Results from mixed logit models of DCE data from Central municipality and Surrounding municipality**

| **Attribute levels^a^** | **Central municipality** | | | **Surrounding municipality** | | |
| --- | --- | --- | --- | --- | --- | --- |
|  | **Mean (SE)** | **SD (SE)** | **WTP (95% confidence interval)** | **Mean (SE)** | **SD (SE)** | **WTP (95% confidence interval)** |
| **Service mode** | | |  |  |  |  |
| General service (ref) | -0.014 (0.036) |  |  | -0.015 (0.035) |  |  |
| Specialized service | 0.014 (0.037) | 0.257 (0.067) ^*^ | 2.41 (-10.33, 15.15) | 0.015 (0.035) | 0.044 (0.207) | 2.37 (-8.44, 13,18) |
| **Medical treatment type** | | | |  |  |  |
| Traditional Chinese Medicine service (ref) | -0.539 (0.096) ^*^ |  |  | -0.525 (0.091) ^*^ |  |  |
| Modern medicine service | 0.393 (0.090) ^*^ | 1.393 (0.109) ^*^ | 69.17 (36.87, 101.46) | 0.420 (0.084) ^*^ | 1.230 (0.097) ^*^ | 66.24 (39.00, 93.48) |
| Integrated medicine service | 0.146 (0.057) ^*^ | 0.303 (0.130) ^*^ | 25.81 (6.48, 45.13) | 0.105 (0.054) ^*^ | 0.005 (0.134) | 16.62 (-0.22, 33.46) |
| **Traveling time to healthcare facility** | | | |  |  |  |
| ≤30mins (ref) | 0.154 (0.037) ^*^ |  |  | 0.369 (0.047) ^*^ |  |  |
| >30mins | -0.154 (0.038) ^*^ | 0.328 (0.056) ^*^ | -27.19 (-40.24, -14.33) | -0.369 (0.048) ^*^ | 0.565 (0.059) ^*^ | -58.29 (-74.18, -42.40) |
| **Type of the physician** | | | |  |  |  |
| Junior health care practitioner (ref) | -0.040 (0.045) |  |  | -0.030 (0.047) |  |  |
| Senior health care practitioner | 0.040 (0.043) | 0.522 (0.058) | 7.01 (-7.61, 21.63) | 0.030 (0.046) | 0.540 (0.060) ^*^ | 4.76 (-9.40, 18.92) |
| **OOP (CNY)** | -0.006 (0.001) ^*^ |  |  | -0.006 (0.001) ^*^ |  |  |
| **Model diagnostics** |  |  |  |  |  |  |
| Number of respondents | 349 |  |  | 331 |  |  |
| Number of observations | 5584 |  |  | 5,296 |  |  |
| Log likelihood | -1573.948 ^*^ |  |  | -1459.333 ^*^ |  |  |

Abbreviation: Mean, coefficient means; SE, standard error; SD, standard deviation; WTP, willingness to pay; ref, reference.

a Coefficients of the reference levels are calculated as the negative sum of the coefficients of the other levels of the attribute.

^*^*p* < 0.05.

**Appendix 6**

**Table S6. WTP in the context of different policy-relevant scenarios**

| **Attribute levels in each scenario** | **Base scenario**  **(Tertiary hospital)** | **Scenario 1**  **(TCM in tertiary hospital)** | **Scenario 2**  **(Integrated medicine in tertiary hospital)** | **Scenario 3**  **(TCM in community clinic)** | **Scenario 4**  **(Integrated medicine in community clinic)** | **Scenario 5**  **(TCM in community clinic by experts)** | **Scenario 6**  **(Integrated medicine in community clinic by experts)** |
| --- | --- | --- | --- | --- | --- | --- | --- |
| Service mode | specialized service | specialized service | specialized service | general service | general service | general service | general service |
| Medical treatment type | modern medicine | TCM | integrated medicine | TCM | integrated medicine | TCM | integrated medicine |
| Traveling time to healthcare facility | traveling time >30mins | traveling time >30mins | traveling time >30mins | traveling time ≤30mins | traveling time ≤30mins | traveling time ≤30mins | traveling time ≤30mins |
| Type of the physician | senior healthcare practitioner | senior healthcare practitioner | senior healthcare practitioner | junior healthcare practitioner | junior healthcare practitioner | senior healthcare practitioner | senior healthcare practitioner |
| WTP (CNY) | 32.69 | -122.86 | -15.45 | -52.44 | 54.96 | -49.11 | 67.30 |
| 95%CI lower bound | 7.37 | -153.41 | -35.52 | -78.49 | 33.14 | -67.00 | 46.78 |
| 95%CI upper bound | 58.00 | -92.30 | 4.62 | -26.40 | 76.79 | -13.22 | 87.81 |
